# Global Analysis of Ankyrin Repeat Domain C3HC4-Type RING Finger Gene Family in Plants

**DOI:** 10.1371/journal.pone.0058003

**Published:** 2013-03-13

**Authors:** Xiaowei Yuan, Shizhong Zhang, Shiyang Liu, Mingli Yu, Hongyan Su, Huairui Shu, Xinzheng Li

**Affiliations:** 1 National Key Laboratory of Crop Biology, Shandong Agricultural University, Tai-An, Shandong, China; 2 National Research Center for Apple Engineering and Technology, Shandong Agricultural University, Tai-An, Shandong, China; 3 Ludong University, Yantai, Shandong, China; Cankiri Karatekin University, Turkey

## Abstract

Ankyrin repeat (ANK) C3HC4-type RING finger (RF) genes comprise a large family in plants and play important roles in various physiological processes of plant life. In this study, we identified 187 ANK C3HC4-type RF proteins from 29 species with complete genomes and named the ANK C3HC4-type RF proteins the XB3-like proteins because they are structurally related to the rice (*Oryza sativa*) XB3. A phylogenetic relationship analysis suggested that the *XB3*-like genes originated from ferns, and the encoded proteins fell into 3 major groups. Among these groups, we found that the spacing between the metal ligand position 6 and 7, and the conserved residues, which was in addition to the metal ligand amino acids, in the C3HC4-type RF were different. Using a wide range of protein structural analyses, protein models were established, and all XB3-like proteins were found to contain two to seven ANKs and a C3HC4-type RF. The microarray data for the *XB3*-like genes of *Arabidopsis*, *Oryza sative*, *Zea mays* and *Glycine max* revealed that the expression of *XB3*-like genes was in different tissues and during different life stages. The preferential expression of *XB3*-like genes in specified tissues and the response to phytohormone and abiotic stress treatments of *Arabidopsis* and *Zea mays* not only confirmed the microarray analysis data but also demonstrated that the XB3-like proteins play roles in plant growth and development as well as in stress responses. Our data provide a very useful reference for the identification and functional analysis of members of this gene family and also provide a new method for the genome-wide analysis of gene families.

## Introduction

Ankyrin repeat (ANK), which is a 33-residue motif in proteins consisting of two alpha helices separated by loops [1], is one of the most common protein domains and is widely distributed in organisms ranging from viruses to human [2]. This domain was initially discovered in the two yeast cell-cycle regulators Swi6/Cdc10 [3] and in the *Drosophila* signalling protein Notch [4],and was named after the discovery of 24 copies of this sequence in the cytoskeletal protein ankyrin [5]. The ANK, which is known to mediate protein-protein interactions [6], has been found in numerous proteins with diverse functions [7], [8].

The RING finger (RF) domain was first identified in a protein encoded by the *Really Interesting New Gene* [9] and was subsequently found in numerous key regulatory proteins [10], [11]. RF is a member of the zinc finger domain protein family, which was first identified as a DNA-binding motif in the transcription factor TFIIIA from *Xenopus laevis* [12]. Subsequently, this domain was identified as functionally binding to RNA [13], protein or lipid substrates [14]. The RING-type is characterised by the presence of a cysteine-rich domain that coordinates two zinc atoms. In addition to the two canonical RING types (C3H2C3 or C3HC4), additional types of modified RING domains, known as RING-V, RING-D, RING-S/T, RING-G and RING-C2, were characterised on the basis of the spacing between their metal ligands or by the different substitutions at one or more of the metal ligand positions [15].

Many RF-containing proteins have E3 ligase role in ubiquitination reaction [15], [16], [17]. The multi-step ubiquitination reaction is initiated by the ATP-dependent binding of ubiquitin, 76-amino acid polymers, to the E1 activation enzyme. The activated ubiquitin is then transferred to an E2 conjugating enzyme, which binds an E3 ligase that facilitates the transfer of ubiquitin to the substrate [18], [19]. These three steps are repeated to attach multiple ubiquitin molecules to substrates [20]. This E3 enzyme is responsible for recruiting the target protein for ubiquitination and, hence, confers specificity to the pathway. In fact, the *Arabidopsis* genome encodes only two E1 isoforms [21], approximately 40 E2 enzymes, and over 1, 400 putative E3 ligases [22]. The existence of such a large number of E3 ligases suggests a high specificity of their target recognition, underscoring their importance for downstream signalling pathways. Among the predicted E3 ligases, 469 proteins contain 477 RING domains [23], which may function as the substrate-binding domain of these E3 ligases [24].

XB3 (XA21-binding protein 3), an E3 ubiquitin ligase, is a substrate for the XA21 serine and threonine kinase and is necessary for the full accumulation of the XA21 protein and for Xa21-mediated immunity [25]. XB3 contains an ANK domain and a RF motif that are sufficient for its interaction with the kinase domain of XA21 and for its E3 ubiquitin ligase activity, respectively [25]. In *Oryza sativa*, the other ANK C3HC4-type RF proteins have been named XBOS [25]. To date, studies demonstrate that the transcription levels of XB3, XBOS31, XBOS32 and XBOS35 can be regulated by hormones and stress [26], [27].

In *Arabidopsis*, five of the ANK C3HC4-type RF proteins have been named XBAT because they are structurally related to the *Oryza sativa* XB3 [28]. XBAT32 and XBAT35 proteins have currently been assigned biological roles. XBAT32 positively regulates lateral root development [28] via the degradation of the ethylene biosynthetic enzyme 1-aminocyclopropane-1-carboxylate synthase 7 [29], thus down-regulating ethylene biosynthesis [30], and may also be implicated in ethylene-mediated responses to abiotic stresses, such as high salinity [31]. In addition, XBAT35 defines a novel player in ethylene signalling that is involved in negatively regulating apical hook curvature, with alternative splicing controlling the dual targeting of this E3 ubiquitin ligase to the nuclear and cytoplasmic compartments [32].

Here, we named a subgroup of the ANK C3HC4-type RF proteins the XB3-like proteins, which contain two to seven N-terminal ANK repeats and a conserved C3HC4-type RF domain. Recent draft genome sequences for plants offer the opportunity to investigate the *XB3*-like genes of plants using genomes that have only recently been completely sequenced. The objective of this study was to identify the complete set of *XB3*-like genes in sequenced plants using a bioinformatics approach. We first identified putative XB3-like proteins in 29 species,then analysed the phylogenetic relationships and structure of these proteins. We analysed the expression patterns of *XB3*-like genes in *Arabidopsis*, *Oryza sative*, *Zea mays* and *Glycine max* using microarray data and surveyed the expression patterns of the *XB3*-like genes in *Arabidopsis* and *Zea mays* as well as their responses to four phytohormones (6-BA, IAA, SA and ABA) and three abiotic stress mimis (NaCl, PEG and mannitol) treatments using real-time PCR. The results constitute a foundation for further functional analyses of each member of this gene family and also provide a new method for analysing gene families in multiple species.

## Materials and Methods

### Identification of *XB3*-like genes in plants

To identify members of the *XB3*-like gene family in plants, we collected the known *XB3*-like genes in *Arabidopsis* and *Oryza sative* and analysed the domains of the XB3-like peptide sequences using PFAM [33] and SMART tools [34]. Three different approaches were then performed. First, all of the known *Arabidopsis* and *Oryza sative XB3*-like gene sequences were used as query sequences to perform multiple database searches against proteome and genome files downloaded from the Phytozome database (http://www.phytozome.net/) [35]. Stand-alone versions of BLASTP and TBLASTN (http://blast.ncbi.nlm.nih.gov), which are available from NCBI, were used with an e-value cutoff of 1e-003 [36], [37]. All protein sequences derived from the collected candidate *XB3*-like genes were examined using the domain analysis programs PFAM (http://pfam.sanger.ac.uk/) and SMART (http://smart.embl-heidelberg.de/) with the default cutoff parameters. Second, we analysed the domains of all plant peptide sequences using a Hidden Markov Model (HMM) [38] analysis with PFAM searching. We then obtained the sequences with the PF12796 and PF13920 PFAM number, which contained a typical ANK domain and a C3HC4-type RING domain, from the plant genome sequences using a Perl-based script. Third, we analysed the domains of all the plant peptide sequences using SMART searching and selected the sequences with an ANK domain (SM00248) and RF structure (SM00184). Finally, all protein sequences were compared with known XB3-like proteins using ClustalX (http://www.clustal.org/) to verify the sequences that were candidate XB3-like proteins [39]. The isoelectric points and molecular weights of XB3-like proteins were obtained with the help of proteomics and sequence analysis tools on the ExPASy Proteomics Server (http://expasy.org/) [40].

### The phylogenetic analysis of XB3-like proteins in plants

XB3-like proteins sequences were aligned using the ClustalX program with BLOSUM30 as the protein weight matrix. The MUSCLE program (version 3.52) was also used to perform multiple sequence alignments to confirm the ClustalX results (http://www.clustal.org/) [41]. Phylogenetic trees of the XB3-like protein sequences were constructed using the neighbour-joining (NJ) method of the MEGA5 program (http://www.megasoftware.net/) using the p-distance and complete deletion option parameters [42]. The reliability of the obtained trees was tested using a bootstrapping method with 1000 replicates. The images of the phylogenetic trees were drawn using MEGA5.

### Expression analyses of the *XB3*-like genes using GENEVESTIGATOR

Microarray expression data from various datasets were obtained using GENEVESTIGATOR (https://www.genevestigator.com/gv/) with the *Arabidopsis*, *Oryza sative*, *Zea mays* and *Glycine max* Gene Chip platform [43].

### Plant material and growth conditions

Columbia 0 (*Arabidopsis thaliana*) and B73 (*Zea mays*) were used for this study. Unless stated otherwise, the seed germination and plant growth conditions were according to Guo et al [44]. For the phytohormone treatments and abiotic stress assays, 12-day-old (*Arabidopsis*) or 5-day-old (*Zea mays*) wild type seedlings were transferred to liquid Murashige and Skoog (MS) medium [45] and supplemented with different treatments (or solvent control) for 6 h with gentle shaking. The plant material was then frozen in liquid N_2_ and stored at −80°C.

### RNA isolation and real-time PCR

Total RNA was isolated from the frozen tissue using the TRIzol reagent (Invitrogen) following the manufacturer's instructions. RNA was further purified using a Fermentas RNAeasy mini kit. RNA (1 µg) was used as a template for first strand cDNA synthesis using the Super Script First-Strand Synthesis system (Transgen). Real-time PCR was performed using gene-specific primers and the TranStart Green qPCR Super Mix (Transgen). Actin2 [46] and 18S ribosomal RNA genes [47] were used as internal normalisation controls. Fold changes in gene expression were calculated using the ΔCt values. To identify preferentially expressed genes, a student-*t* test was performed. A gene in a given tissue was defined as preferentially expressed only if the expression value of the gene in this tissue was more than 2-fold and had a *P* value less than 0.05 compared to other tissues. Under phytohormone and abiotic treatments, genes that were up or down-regulated more than 1.2-fold and with *P* value less than 0.05 compared to control were considered as differentially expression. Details of the primers used are listed in Table 1.

**Table 1:** The primers used for real-time PCR of *XB3*-like genes in *Arabidopsis* and *Zea mays.*

Gene	Primers for real-time PCR (5′-3′)
*XBAT31*	Forward: AGGCTTTAATGGAGGCTAACAGG
	Reverse: GAGAAAGAGGGTGATGGTAAGGAA
*XBAT32*	Forward: CGAAACTGGCTGGAAGAAAT
	Reverse: CAAACGGCACAAGGGTCA
*XBAT33*	Forward: GAAGCACGCCTTTACACTATG
	Reverse: ATTTGGTGAAAGCAACGGT
*XBAT34*	Forward: TCATTTCTCAGGCGAGGCGT
	Reverse: CATTGGCTGTGGAACTCCTTTAC
*XBAT35*	Forward: TGCTTATCGTCCTGGTCG
	Reverse: ACATTGCTAAACCCTTTGACT
*ACT2*	Forward: TTGTGCTGGATCTGGTGATG
	Reverse: CGCTCTGCTGTTGTGGTG
*ZmaXB31*	Forward: GCAGCAGCCTCAACTCGC
	Reverse: GGACACGTTGCATCCGAAA
*ZmaXB32*	Forward: GAATCGGCACAAGCAGACG
	Reverse: CTGGGAGTCGAACATGAGGATAT
*ZmaXB33*	Forward: CCCAACCCGACGACCCTG
	Reverse: CAAAGAATGACTGGGGTAATGAGC
*ZmaXB34*	Forward: ACCAGCAGGTCAACTACGGC
	Reverse: CATACAGGCAAGCATCAGGG
*ZmaXB36*	Forward: GCTGGAACCCGACGCCAAATC
	Reverse: CGACGGCAACGCATTCTTAGTCC
18S	Forward: GATACCGTCCTAGTCTCAACC
	Reverse: GCCTTGCGACCATACTCC

## Results

### Identification of *XB3*-like genes in plants

**Figure 1:**
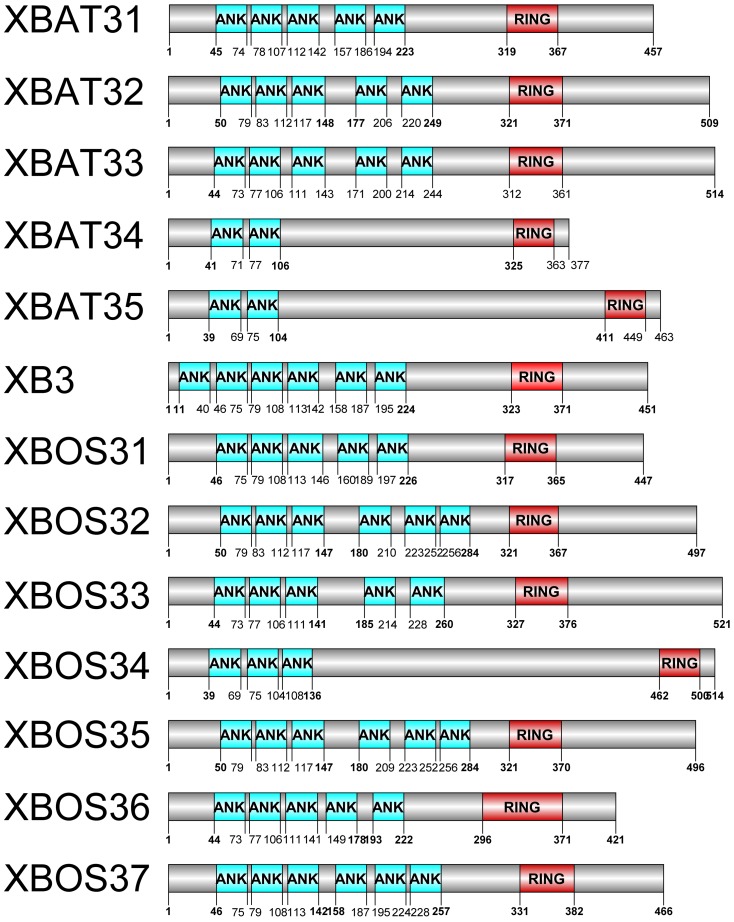
The structures of XB3-like proteins in *Arabidopsis* and *Oryza sative*. The domains of the XB3-like protein were analysed using SMART searching, and the image was produced using DOG2.0.The gray box in the protein structure diagram represents the XB3-like proteins, the blue and the red boxes represent the ANK domain and C3HC4-type RF domain, respectively. The numbers under the proteins indicate the position of the domain.

To identify *XB3*-like genes in plants, we first collected the known *XB3*-like genes from *Arabidopsis* (*XBAT31, XBAT32, XBAT33, XBAT34, XBAT35*) and *Oryza sative* (*XB3, XBOS31, XBOS32, XBOS33, XBOS34, XBOS35, XBOS36*) and analysed the XB3 domains and XBAT and XBOS peptides using the PFAM and SMART tools. Our result demonstrated that all twelve proteins contained a conserved C3HC4 RF domain and no fewer than two ANK domains (XBAT34 and XBAT35 contain two ANK domains; XBOS34 contains three ANK domains; XBAT31, XBAT32, XBAT33, XBOS31, XBOS33 and XBOS36 contain five ANK domains; and XB3, XBOS32 and XBOS35 contain six ANK domains; Fig. 1). We then used bioinformatics methods to gather extensive information regarding this family. A total of 187 genes encoding 187 XB3-like proteins were identified as potential members of the *XB3*-like gene family within the 29 plant genomes (no *XB3*-like gene in *Chlamydomonas reinhardtii* or *Volvox carteri*) that have been completely sequenced (Phytozome database: http://www.phytozome.net/; Table S1). Because there was no standard annotation assigned to these newly identified plant genes (not including *Arabidopsis* and *Oryza sative*), we assigned each of them an identity based on the gene identifier. Interestingly, a new *XBOS* gene (Os03g16780) was identified for the first time in *Oryza sative* using this method and named *XBOS37* (Fig. 1).

**Figure 2:**
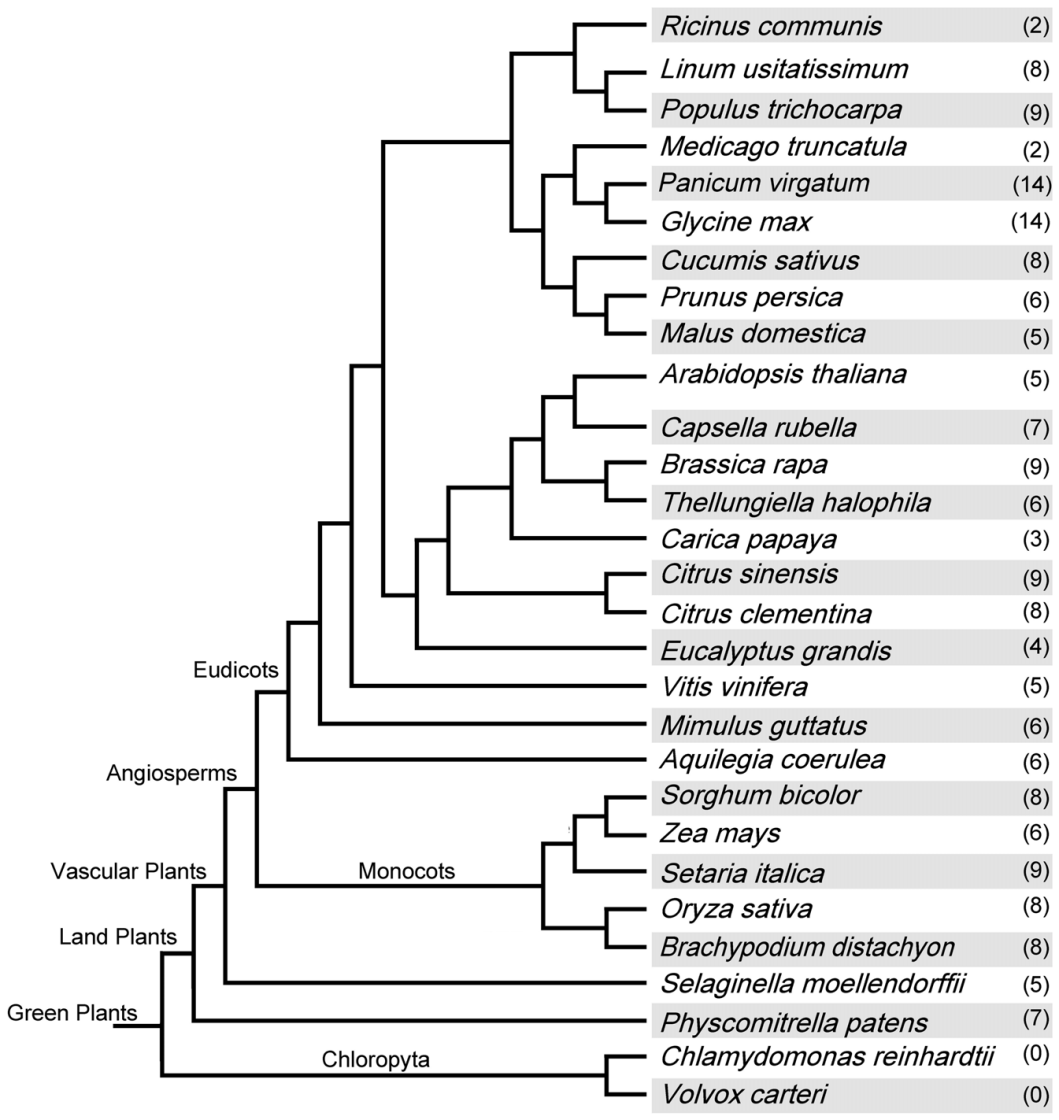
The phylogenetic relationships of the plants with completely sequenced genomes. The number in parentheses corresponds to the number of *XB3*-like genes in each species.

In this study, we observed that the *XB3*-like gene family originated from the ferns and were identified in 27 species of land plants. The results suggested that the function of the XB3-like proteins may be involved in the morphological character of land plants and adaptations to survival in certain environments. Additionally, among the land plants, there are 2 (*Ricinus communis* and *Medicago truncatula*) to 14 (*Panicum virgatum* and *Glycine max*) *XB3*-like genes in every species, and most species possess more than five XB3-like proteins (Fig. 2). The protein length of the *XB3*-like gene family is from 327 aa (GmaXB32) to 632 aa (AcoXB35), and the isoelectric point is from 5.31 (PpaXB31) to 9.50 (SmoXB32) (Table S1).

### Phylogenetic relationships between the *XB3*-like family genes

**Figure 3:**
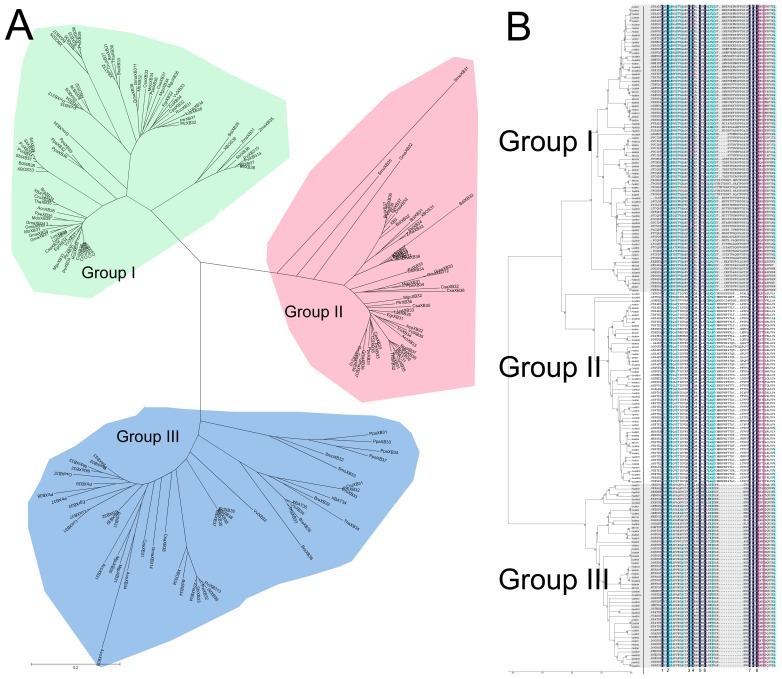
Phylogenetic relationships between the *XB3*-like genes. (A) The phylogenetic tree of the plant XB3-like proteins. The amino acid sequences of the plant XB3 proteins were aligned using MUSCLE, and the phylogenetic tree was constructed using the neighbour-joining method in the MEGA 5 software. Each node has a number that indicates the bootstrap value for 1000 replicates. The scale bar represents 0.1 substitutions per sequence position. (B) Multiple sequence alignment of representative RING domains. Dashes denote the gaps introduced to maximise the alignment, asterisks indicate conserved residues and the black dots indicate residues conserved in all RING types.

To clarify the phylogenetic relationship among the *XB3*-like genes and to infer the evolutionary history of the gene family, the full-length protein sequences of the XB3-like family members in plants were used to construct a joint unrooted phylogenetic tree (Fig. 3A), from which it can be observed that the proteins fell into three major groups (group I to group III) with well-supported bootstrap values. Statistically, group I contains 81 members, group II contains 54 members and group III has 52 members. In addition, most plants contain 3 groups of *XB3*-like genes, except for *Ricinus communis* and *Medicago truncatula,* which only contains group I *XB3*-like genes.

Interestingly, we found that the C3HC4-type RF domain was conserved in each group. Previous studies have revealed that the RF domain forms a distinct so-called cross-brace structure in which metal ligand pairs 1 and 3 coordinate to bind one zinc ion and pairs 2 and 4 bind the second one. This special structure requires that the distance between metal ligands 1 and 2, 3 and 6 as well as 7 and 8 should be conserved while the distance between metal ligands 2 and 3, 6 and 7 can vary. An alignment of all C3HC4-type RF domains was performed (Fig. 3B). All three groups of XB3-like proteins contained two amino acids between metal ligands 1 and 2 as well as 7 and 8 (except for VviXB35), 11 amino acids between metal ligands 2 and 3, and 7 amino acids between metal ligands 3 and 6. However, the number of residues between metal ligands 6 and 7 was from 19 to 23 in group I, 21 to 24 in group II and 11 in group III, respectively (Table S2). In addition to the conserved metal ligand positions, a previous study demonstrated that other positions also display a high level of similarity in the RING domain of *Arabidopsis*. Notably, a Pro (P) residue is present right after metal ligand 7 in all three groups of XB3-like proteins, and an Arg (R) residue is present after metal ligand 8 in all identified proteins, except for ThaXB34. Meanwhile, the first residue before metal ligand 2 is always a Val (V) or Ile (I). In addition, the residue before metal ligand 4 is always a Gly (G) in group II proteins. The residue before metal ligand 8 is a Leu (L) in group I, a Phe (F) in group II and a Val (V) or Ile (I) in group III, respectively. Therefore, we hypothesise that proteins in the same group likely perform the same or similar functions in plants.

### Classification of XB3 proteins based on ANK number

**Figure 4:**
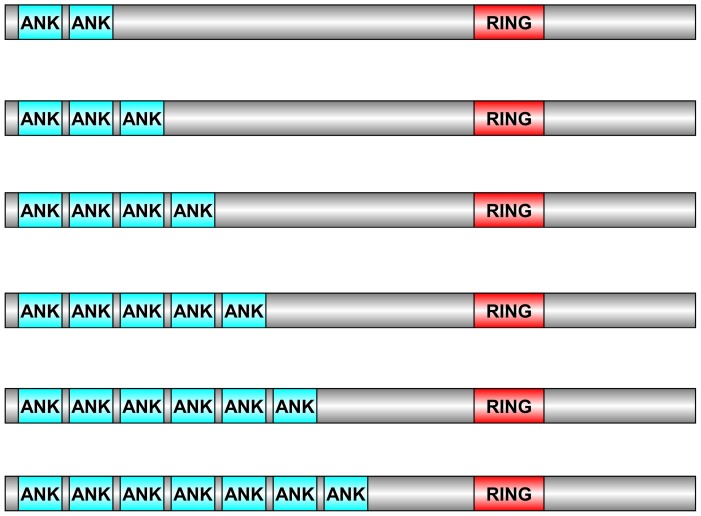
The protein models of XB3-like plant proteins. The image was produced using DOG2.0. The gray box in the protein structure diagram represents the XB3-like proteins, and the blue and the red boxes represent ANK domains and C3HC4-type RF domains, respectively. (B) The classification of XB3-like proteins in plants.

Based on the detailed results from the PFAM and SMART searches, the XB3-like proteins were classified into six groups based on their number of ANKs (Fig. 4A). The number of ANK was from 2 to 6 in group A to F. However, there was no significant difference in the length of the proteins in the six groups. The numbers of members in group A to F were 27, 20, 14, 91, 31 and 4, respectively (Table S1). In accordance with the trend of ANK domains in *Arabidopsis* and *Oryza sative*, the proteins containing five ANK domains are approximately 50% in the gene family. These results suggested that the function of the ANK domain, known to mediate protein-protein interactions, may be related to the number of ANKs.

### Expression profiling of *XB3*-like genes in *Arabidopsis*, *Oryza sative*, *Zea mays* and *Glycine max*

**Figure 5:**
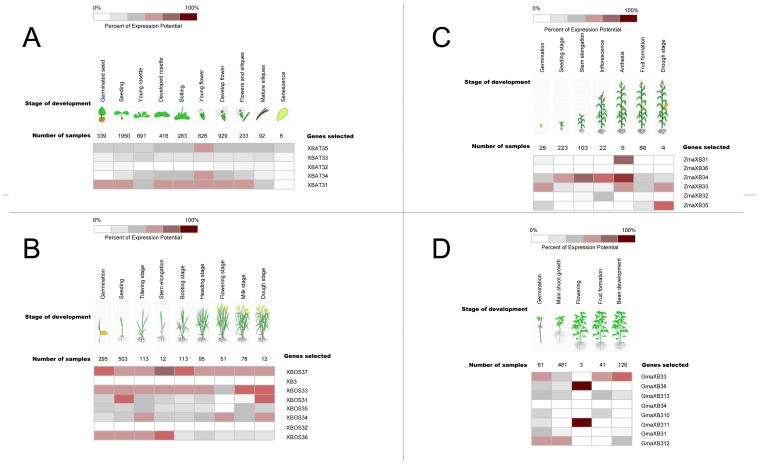
Expression analysis of *XB3*-like genes in *Arabidopsis*, *Oryza sative*, *Zea mays* and *Glycine max*. The dark and light colour shadings represent relative high or low expression levels, respectively, of the *XB3* genes in different tissues. (A) The expression profile of *XB3*-like genes in *Arabidopsis*. (B) The expression profile of *XB3*-like genes in *Oryza sative*. (C) The expression profile of *XB3*-like genes in *Zea mays*. (D) The expression profile of *XB3*-like genes in *Glycine max*.

To investigate the expression profiling of the *XB3*-like gene family in plants, we used bioinformatics methods to gather extensive microarray information regarding this family in the model plant *Arabidopsis* and in other crops (*Oryza sative*, *Zea mays* and *Glycine max*; Fig. 5). The developmental stages selected for the microarray analysis cover the entire life cycle. The sample number indicates the number of microarray analyses, and the dark and light colour shadings represent relative high or low expression levels of the *XB3*-like genes in different tissues, respectively.

In *Arabidopsis,* five members of the *XB3*-like gene family were identified: *XBAT31*, *XBAT32*, *XBAT33*, *XBAT34* and *XBAT35*. In addition to *XBAT32*, four of these members demonstrated extensive expression levels in all of the developmental stages and tissues analysed (Fig. 5A), and *XBAT31* had the highest expression level among the five genes, which had its highest expression in the senescent leaf, stem and flower (Fig. S1). In contrast, we found that the expression of *XBAT32* was regionally in the root stele and anther. In *Oryza sative*, in addition to *XB3*, seven homologous genes were identified. Among these eight genes, *XBOS33* and *XBOS37* had the highest expression levels during the entire life cycle and in different tissues (Fig. S2). Additionally, high expression signals were detected for *XBOS36* during stem elongation and for *XBOS31* during the seedling and dough stage. In *Zea mays,* among the five *XB3*-like genes, *ZmaXB34* had the highest expression level during the entire life cycle. In addition, the expression of *ZmaXB31* was highest during the anthesis stage, and *ZmaXB35* had its highest expression during the dough stage. In *Glycine max,* fourteen members of the *XB3*-like gene family were identified. *GmaXB38* and *GmaXB311* had the same expressional patterns and had their highest expression levels during the flowering stage, especially in flowers (Fig. S3). In addition, although *GmaXB33* had a higher expression level than that of *GmaXB313*, the expression patterns of *GmaXB33* and *GmaXB313* were similar.

**Figure 6:**
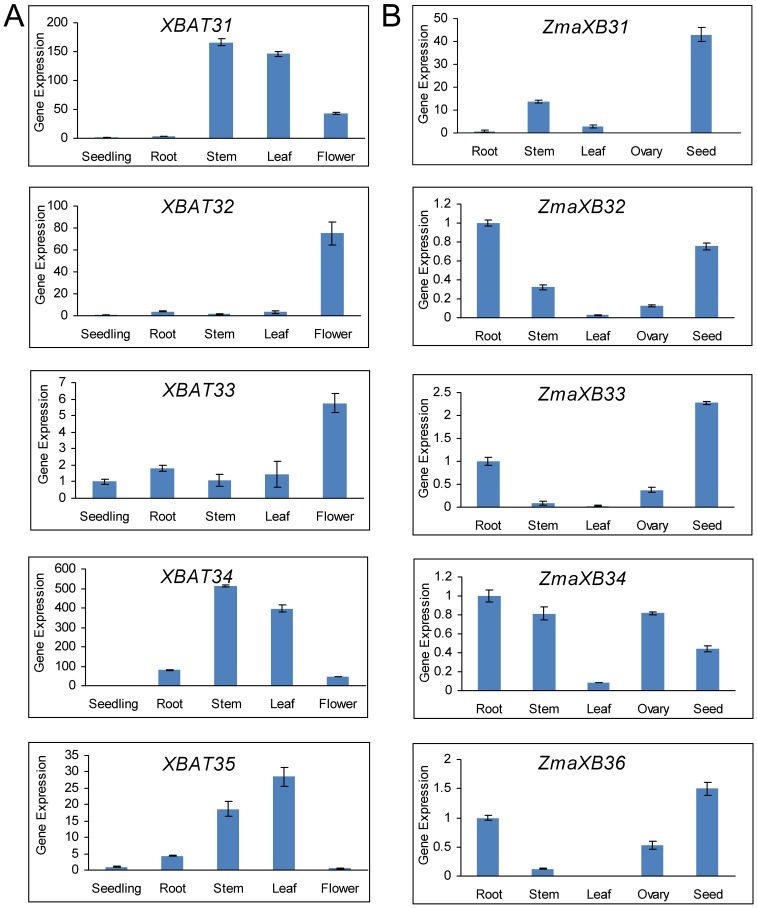
Expression profiles of *XB3*-like genes in *Arabidopsis* and *Zea mays*. (A) The spatial expression patterns of *XB3*-like genes in *Arabidopsis* seedlings (12-day-old seedlings). Root means of two-week-old seedlings. Stem means of six-week-old plants. Leaf means of four-week-old plants. Flower means opening flower. Representative experiments are shown and were performed three times. Each bar represents a mean±SEM (n = 3). (B) The spatial expression profile of *XB3*-like genes in *Zea mays*. Root means of four-week- old seedlings. Stem means of four-week-old seedlings. Leaf means of four-week-old plants. Ovary means one-day old ovary. Seed means 9-day-old seed. Representative experiments are shown and were performed three times. Each bar represents a mean±SEM (n = 3).

With the aim of revealing the *XB3*-like gene expression features, an analysis of preferential expression was performed using real-time PCR with gene-specific primers in *Arabidopsis* and *Zea mays*. In *Arabidopsis*, the predominant expression of *XBAT31*, *XBAT34* and *XBAT35* was in stem and leaf while *XBAT32* and *XBAT33* had their highest expression levels in flower (Fig. 6A). In *Zea mays*, *ZmaXB34* was highly and extensively expressed (Fig. S4). High expression levels of *ZmaXB32*, *ZmaXB33* and *ZmaXB36* were observed in the root and seed while *ZmaXB31* had its highest expression only in seed (Fig. 6B). These results demonstrated that the expression of *XB3*-like genes was extensive during different development stages and in different tissues.

### Responses of *XB3*-like genes to phytohormones and abiotic stress in *Arabidopsis* and *Zea mays*

**Figure 7:**
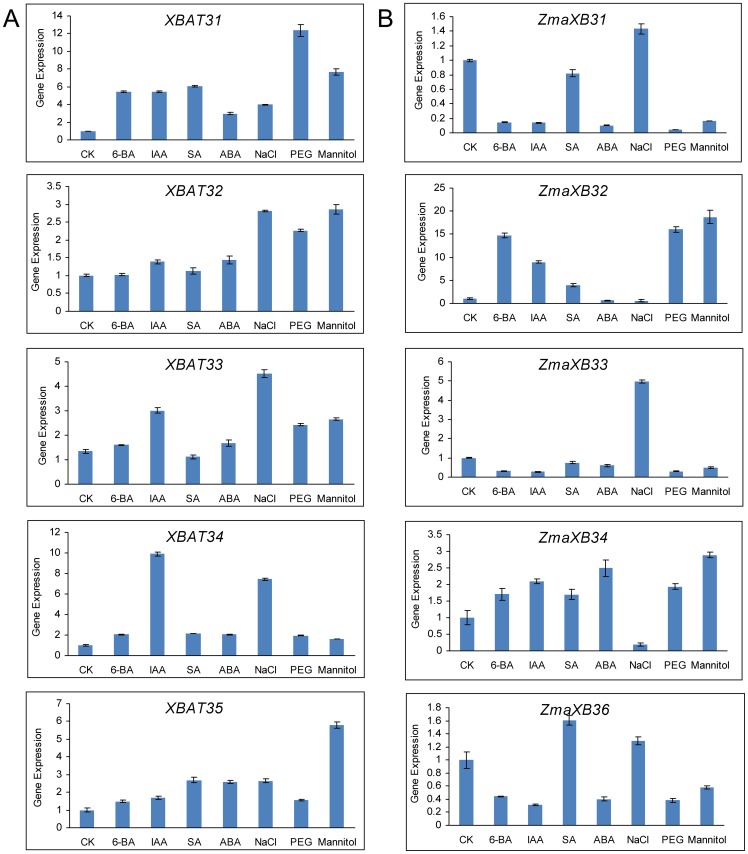
The expression profiles of some *XB3*-like genes are responsive to phytohormones and mimic abiotic stress. Twelve-day-old (*Arabidopsis*) or 5-day-old (*Zea mays*) wild type seedlings were transferred to liquid MS media supplemented with 5 µM 6-BA, 5 µM IAA, 100 µM SA, 100 µM ABA, 100 mM NaCl, 300 mM mannitol or 15% PEG 6000 (or solvent control) for 6 h with gentle shaking. Representative experiments are shown and were performed three times. Each bar represents a mean±SEM (n = 3).

Phytohormones play a critical role in plant growth and development. To investigate the potential function of the *XB3*-like gene family in plant, we surveyed the responses of *XB3*-like genes to phytohormones in *Arabidopsis* and *Zea mays* using real-time PCR. In *Arabidopsis*, all of the *XB3*-like genes were up-regulated by IAA (indole-3-acetic acid) and ABA (abscisic acid). In addition, four genes were induced by 6-BA (6-benzylaminopurine) (*XBAT31*, *XBAT33*, *XBAT34* and *XBAT35*) and SA (salicylic acid) (*XBAT31*, *XBAT32*, *XBAT34* and *XBAT35*) (Fig. 7A). However, all five examined genes were down-regulated by ABA, and three genes (ZmaXB31, ZmaXB33 and ZmaXB36) were down-regulated by 6-BA and IAA in *Zea mays* (Fig. 7B). Therefore, the results demonstrated that phytohormones affect the expression of *XB3*-like genes and suggest that these proteins may play roles in plant growth and development.

Previous reports have revealed that ABA, as stress a hormone, mediates the plant's response to stress by its rapid accumulation, thus helping plant survival [48], [49]. All of the ten genes mentioned above were affected by ABA, and we speculate that *XB3*-like genes may be involved in the signalling pathways triggered by abiotic stresses. To reveal the responses of these genes to different stresses, real-time PCR analysis was performed using the total RNA from the leaves of seedling treated with NaCl, PEG and mannitol (Fig. 7). The results demonstrated that all of the *XB3*-like genes in *Arabidopsis* were induced by NaCl, PEG and mannitol. In *Zea mays*, the expression of *ZmaXB31*, *ZmaXB33* and *ZmaXB36* were induced by NaCl and suppressed by PEG and mannitol. On the contrary, the transcription of *ZmaXB32* and *ZmaXB34* was suppressed by NaCl and induced by PEG and mannitol. These results suggest that the functions of *XB3*-like genes may be involved in responses to abiotic stresses in plants.

## Discussion

With the development of comparative genomics, it is now possible to analyse proteins from the same gene family among different species. To clarify the phylogenetic relationship among the *XB3*-like genes and to infer functions as well as the evolutionary history of this gene family, we identified 187 genes in 27 land plants with completely sequenced genomes. Notably, the XB3-like proteins originated from the ferns and are conserved in land plants (Fig. 2). These results infer that the structure of these proteins has been conserved and that these proteins are essential for plant survival on the land. In addition, we found that all of the XB3-like proteins fall into three groups in which there is a conserved spacing between the metal ligand positions and the conserved residues in addition to the metal ligand amino acids in the C3HC4-type RF, which are different among the three groups (Fig. 3). A previous study demonstrated that RF can bind to DNA, RNA, proteins or lipid substrates [14]. Therefore, we speculated that the binding substrates of the proteins in the same group maybe similar.

Because XB3-like proteins appear to be conserved in terms of structure rather than in function, knowing the expression profiles of some *XB3*-like genes may provide clues to the function of *XB3*-like genes. Through the use of expression profile analyses in *Arabidopsis, Oryza sative*, *Zea mays* and *Glycine max*, the accumulation of *XB3*-like gene transcripts was demonstrated during different developmental stages and in different tissues (Fig. 5, 6). Therefore, the variability in the expression patterns of genes in the same family indicated that their roles might not be redundant and that these genes, which are preferentially expressed in specific tissues, may deserve further investigation for their functions. In addition, crops and the model plant *Arabidopsis* were used in this expression analysis. Therefore, the results from this study will be useful for further studying crop production.

The proteins of the *XB3*-like gene family, as identified in different plant species, are involved in various developmental processes, including signalling pathways, stress responses, and plant defences [25], [29], [32]. As is known, phytohormones play important roles in plant growth and development as well as in stress tolerance [50], [51], [52], [53], [54]. Some *XB3*-like genes in plants are affected by phytohormones and play roles in stress responses. For example, XB3 plays a role in resistance against *Xanthomonas oryzae* pv. *Oryzae* [25]. The expression of *XBOS31*, *XBOS32* and *XBOS35* can be regulated by hormones as well as stresses. Auxin can induce the expression of *XBAT32* [28]. The transcription level of *XBAT35* is up-regulated in response to exogenous glucose and NaCl as well as heat and cold stresses [32]. In addition, the functions of other ANK-RF protein were demonstrated. KEEP ON GOING (KEG), a RING E3 ligase essential for *Arabidopsis* growth and development, is involved in ABA signalling [55]. *MjXB3*, which is highly expressed in the petals of senescing four o′clock (*Mirabilis jalapa*) flowers, is involved in the coordination of the senescence program [56]. The transcription of *AdZFP1*, which was isolated from drought-tolerant *Artemisia desertorum*, is induced by exogenous ABA and also by salinity, cold and heat, to some extent [57]. Therefore, knowing the response of *XB3*-like genes to phytohormones and mimicking abiotic stress treatments may provide clues to their function. Through our transcriptional analysis of some of the *XB3*-like genes, we found that the expression of all the examined genes was affected by many phytohormones (Fig. 7). This result suggests that the XB3-like proteins likely play roles in plant growth and development. In addition, all ten genes are affected by ABA, which is a stress hormone [58], [59], specifically abiotic stress [60]. Interestingly, we found that the expression of *XB3*-like genes was induced by salt and mimicked drought stress in *Arabidopsis*. However, in *Zea mays*, the expression of some *XB3*-like genes was induced by mimicking drought stress and suppressed by salt stress, and some *XB3*-like genes were induced by salt stress and suppressed by mimicking drought stress. Previous studies have revealed that salt and drought stress signal a transduction that consists of the ionic and osmotic homeostasis signalling pathway [61], [62]. Therefore, our results infer that *XB3*-like genes are involved in responses to abiotic stresses, likely in different pathways in plants.

In conclusion, the preferential expression in specified tissues and the response to phytohormones and abiotic stress treatments of the *XB3*-like genes provide clues to the roles of these genes in signalling, growth and development. The systematic sequence analysis and expression profiles of the *XB3*-like genes will serve as a very useful reference for more detailed functional analyses and will be helpful in the selection of appropriate candidate genes for further studies and genetic engineering.

## Supporting Information

Figure S1 **Spatial expression analysis of** ***XB3*** **-like genes in** ***Arabidopsis*** **.**
(TIF)

Figure S2 **Spatial expression analysis of** ***XB3*** **-like genes in** ***Oryza sative*** **.**
(TIF)

Figure S3 **Spatial expression analysis of** ***XB3*** **-like genes in** ***Glycine max*** **.**
(TIF)

Figure S4 **Spatial expression analysis of** ***XB3*** **-like genes in** ***Zea mays*** **.**
(TIF)

Table S1 **The** ***XB3-Like*** **genes in plants.**
(DOC)

Table S2 **Number and consensus of each group type C3HC4-RING domain identified in plants.**
(DOC)
