# Supplementary material for: Global Analysis of Ankyrin Repeat Domain C3HC4-Type RING Finger Gene Family in Plants
Source: PLoS One. 2013 Mar 13;8(3):e58003. doi: 10.1371/journal.pone.0058003 (PMC3596331; doi:10.1371/journal.pone.0058003)
Supplement: Table S1 — The XB3-Like genes in plants. (DOC) [file pone.0058003.s005.doc]

Table S1 The *XB3*-like genes in plants.

| Species | Gene name | Gene identifier | PACid | Size(aa) | Mass(Da) | IP | Number of Ank |
| --- | --- | --- | --- | --- | --- | --- | --- |
| *Aquilegia coerulea* | *AcoXB31* | Aquca_017_00738.1 | 22031203 | 561 | 60359.27 | 6.18 | 3 |
| *Aquilegia coerulea* | *AcoXB32* | Aquca_027_00342.1 | 2204527 | 447 | 48410.5 | 8.03 | 5 |
| *Aquilegia coerulea* | *AcoXB33* | Aquca_003_00026.1 | 2204997 | 451 | 48718.45 | 6.94 | 5 |
| *Aquilegia coerulea* | *AcoXB34* | Aquca_034_00354.1 | 2205640 | 489 | 52336.46 | 6.43 | 2 |
| *Aquilegia coerulea* | *AcoXB35* | Aquca_034_00356.1 | 22056442 | 632 | 69471 | 6.26 | 3 |
| *Aquilegia coerulea* | *AcoXB36* | Aquca_013_00388.1 | 22059895 | 512 | 55248.98 | 6.64 | 5 |
| *Arabidopsis thaliana* | *XBAT31* | AT2G28840.1 | 19641385 | 457 | 49609.78 | 8.01 | 5 |
| *Arabidopsis thaliana* | *XBAT34* | AT4G14365.1 | 19648313 | 377 | 41382.67 | 8.15 | 2 |
| *Arabidopsis thaliana* | *XBAT35* | AT3G23280.1 | 19663410 | 463 | 50055.14 | 8.33 | 2 |
| *Arabidopsis thaliana* | *XBAT33* | AT5G07270.1 | 19665812 | 514 | 55347.23 | 6.46 | 5 |
| *Arabidopsis thaliana* | *XBAT32* | AT5G57740.1 | 19668078 | 509 | 54586.1 | 8.42 | 5 |
| *Brachypodium distachyon* | *BdiXB31* | Bradi2g62400.1 | 21805767 | 453 | 47190.59 | 6.67 | 5 |
| *Brachypodium distachyon* | *BdiXB32* | Bradi2g37100.1 | 21805877 | 452 | 48399.93 | 6.59 | 5 |
| *Brachypodium distachyon* | *BdiXB33* | Bradi1g66640.1 | 21818813 | 505 | 54679.25 | 8.97 | 5 |
| *Brachypodium distachyon* | *BdiXB34* | Bradi1g28820.1 | 21821209 | 516 | 55047.74 | 6.72 | 3 |
| *Brachypodium distachyon* | *BdiXB35* | Bradi1g49680.1 | 21822486 | 392 | 39907.26 | 5.59 | 4 |
| *Brachypodium distachyon* | *BdiXB36* | Bradi3g53810.1 | 21827550 | 493 | 53143.65 | 6.24 | 5 |
| *Brachypodium distachyon* | *BdiXB37* | Bradi3g19170.1 | 21829625 | 486 | 52033.97 | 8.78 | 5 |
| *Brachypodium distachyon* | *BdiXB38* | Bradi3g31460.1 | 21829747 | 521 | 55193.69 | 5.92 | 5 |
| *Brassica rapa* | *BraXB31* | Bra002699 | 22694522 | 516 | 55315.65 | 8.10 | 5 |
| *Brassica rapa* | *BraXB32* | Bra028419 | 22690069 | 382 | 42171.39 | 8.17 | 3 |
| *Brassica rapa* | *BraXB33* | Bra020414 | 22690964 | 506 | 53889.2 | 7.17 | 6 |
| *Brassica rapa* | *BraXB34* | Bra009278 | 22692777 | 516 | 55741.63 | 6.40 | 5 |
| *Brassica rapa* | *BraXB35* | Bra014934 | 22701461 | 449 | 48736.66 | 7.91 | 2 |
| *Brassica rapa* | *BraXB36* | Bra014932 | 22701746 | 357 | 39193.31 | 8.75 | 2 |
| *Brassica rapa* | *BraXB37* | Bra011923 | 22702235 | 458 | 49626.69 | 8.03 | 5 |
| *Brassica rapa* | *BraXB38* | Bra000480 | 22712470 | 444 | 48475.4 | 8.33 | 4 |
| *Brassica rapa* | *BraXB39* | Bra035664 | 22719140 | 452 | 48922.76 | 7.18 | 5 |
| *Citrus clementina* | *CclXB31* | clementine0.9_008699m | 19257780 | 488 | 52671.35 | 8.70 | 2 |
| *Citrus clementina* | *CclXB32* | clementine0.9_009360m | 19257781 | 471 | 50439.69 | 7.12 | 2 |
| *Citrus clementina* | *CclXB33* | clementine0.9_010061m | 19260716 | 450 | 48605.31 | 5.60 | 6 |
| *Citrus clementina* | *CclXB34* | clementine0.9_007891m | 19263282 | 510 | 55201.13 | 8.48 | 5 |
| *Citrus clementina* | *CclXB35* | clementine0.9_036023m | 19263483 | 509 | 55280.29 | 6.14 | 2 |
| *Citrus clementina* | *CclXB36* | clementine0.9_007737m | 19275713 | 514 | 55239.26 | 6.31 | 5 |
| *Citrus clementina* | *CclXB37* | clementine0.9_007768m | 19275714 | 514 | 55239.26 | 6.31 | 5 |
| *Citrus clementina* | *CclXB38* | clementine0.9_010311m | 19282218 | 444 | 47980.69 | 6.80 | 6 |
| *Carica papaya* | *CpaXB31* | evm.model.supercontig_44.48 | 16420187 | 487 | 53142.59 | 7.86 | 3 |
| *Carica papaya* | *CpaXB32* | evm.model.supercontig_75.17 | 16425853 | 496 | 53882.49 | 7.17 | 5 |
| *Carica papaya* | *CpaXB33* | evm.model.supercontig_81.163 | 16426923 | 445 | 48230.97 | 6.92 | 6 |
| *Capsella rubella* | *CruXB31* | Carubv10005117m | 20895608 | 361 | 39854.82 | 8.22 | 2 |
| *Capsella rubella* | *CruXB32* | Carubv10005115m | 20895609 | 361 | 39826.72 | 8.22 | 2 |
| *Capsella rubella* | *CruXB33* | Carubv10005114m | 20895610 | 361 | 39853.83 | 8.46 | 2 |
| *Capsella rubella* | *CruXB34* | Carubv10015729m | 20899783 | 465 | 50053.02 | 8.33 | 2 |
| *Capsella rubella* | *CruXB35* | Carubv10023180m | 20904190 | 457 | 49613.69 | 7.80 | 5 |
| *Capsella rubella* | *CruXB36* | Carubv10000735m | 20910124 | 516 | 55530.45 | 6.43 | 5 |
| *Capsella rubella* | *CruXB37* | Carubv10026263m | 20911550 | 508 | 54611.22 | 8.40 | 5 |
| *Cucumis sativus* | *CsaXB31* | Cucsa.063710.1 | 16954998 | 471 | 51734.29 | 8.24 | 5 |
| *Cucumis sativus* | *CsaXB32* | Cucsa.066120.1 | 16955151 | 444 | 48166.17 | 7.07 | 6 |
| *Cucumis sativus* | *CsaXB33* | Cucsa.089800.1 | 16957207 | 511 | 55238.02 | 8.20 | 5 |
| *Cucumis sativus* | *CsaXB34* | Cucsa.166480.1 | 16966171 | 513 | 54884.61 | 6.48 | 5 |
| *Cucumis sativus* | *CsaXB35* | Cucsa.172430.1 | 16966585 | 448 | 48496.23 | 7.01 | 6 |
| *Cucumis sativus* | *CsaXB36* | Cucsa.175400.1 | 16966749 | 438 | 47737.6 | 6.24 | 7 |
| *Cucumis sativus* | *CsaXB37* | Cucsa.255450.1 | 16972398 | 477 | 51455.27 | 6.15 | 3 |
| *Cucumis sativus* | *CsaXB38* | Cucsa.383600.1 | 16982414 | 488 | 52824.08 | 8.52 | 2 |
| *Citrus sinensis* | *CsiXB31* | orange1.1g010335m | 18101176 | 514 | 55241.23 | 6.31 | 5 |
| *Citrus sinensis* | *CsiXB32* | orange1.1g011309m | 18101177 | 490 | 52405.87 | 6.16 | 4 |
| *Citrus sinensis* | *CsiXB33* | orange1.1g014068m | 18101178 | 432 | 46677.58 | 6.49 | 3 |
| *Citrus sinensis* | *CsiXB34* | orange1.1g012618m | 18111681 | 461 | 49720.69 | 5.69 | 6 |
| *Citrus sinensis* | *CsiXB35* | orange1.1g010439m | 18113885 | 511 | 55540.54 | 6.14 | 2 |
| *Citrus sinensis* | *CsiXB36* | orange1.1g010517m | 18113886 | 509 | 55265.23 | 6.14 | 2 |
| *Citrus sinensis* | *CsiXB37* | orange1.1g010851m | 18113887 | 500 | 54256 | 6.09 | 2 |
| *Citrus sinensis* | *CsiXB38* | orange1.1g010920m | 18113888 | 498 | 54224 | 5.93 | 2 |
| *Citrus sinensis* | *CsiXB39* | orange1.1g040660m | 18120528 | 488 | 52631 | 8.59 | 2 |
| *Eucalyptus grandis* | *EgrXB31* | Eucgr.B02375.1 | 23567503 | 446 | 47776 | 7.52 | 7 |
| *Eucalyptus grandis* | *EgrXB32* | Eucgr.C01246.1 | 23570814 | 510 | 55120 | 8.66 | 5 |
| *Eucalyptus grandis* | *EgrXB33* | Eucgr.H03040.1 | 23592362 | 486 | 52392 | 7.46 | 3 |
| *Eucalyptus grandis* | *EgrXB34* | Eucgr.K00236.1 | 23601692 | 512 | 54837 | 6.14 | 5 |
| *Glycine max* | *GmaXB31* | Glyma16g32090.1 | 16303583 | 505 | 54125 | 6.46 | 5 |
| *Glycine max* | *GmaXB32* | Glyma08g42740.1 | 16273620 | 327 | 36074 | 8.90 | 5 |
| *Glycine max* | *GmaXB33* | Glyma08g47310.1 | 16274143 | 439 | 47539 | 6.80 | 6 |
| *Glycine max* | *GmaXB34* | Glyma09g26560.1 | 16276320 | 505 | 54160 | 6.61 | 5 |
| *Glycine max* | *GmaXB35* | Glyma10g38270.1 | 16281383 | 518 | 55491 | 6.48 | 5 |
| *Glycine max* | *GmaXB36* | Glyma11g14900.1 | 16283930 | 448 | 48313 | 7.25 | 6 |
| *Glycine max* | *GmaXB37* | Glyma12g06850.1 | 16286669 | 448 | 48438 | 7.53 | 6 |
| *Glycine max* | *GmaXB38* | Glyma13g01480.1 | 16289334 | 509 | 54971 | 7.2 | 5 |
| *Glycine max* | *GmaXB39* | Glyma13g41040.1 | 16293357 | 452 | 48554 | 7.26 | 7 |
| *Glycine max* | *GmaXB310* | Glyma15g04410.1 | 16297608 | 445 | 47867 | 6.81 | 7 |
| *Glycine max* | *GmaXB311* | Glyma17g07600.1 | 16304766 | 511 | 55304 | 7.73 | 5 |
| *Glycine max* | *GmaXB312* | Glyma18g38610.1 | 16309914 | 443 | 48033 | 6.69 | 6 |
| *Glycine max* | *GmaXB313* | Glyma20g29590.1 | 16317277 | 513 | 54934 | 6.61 | 5 |
| *Glycine max* | *GmaXB314* | Glyma20g35150.1 | 16317921 | 440 | 47543 | 8.31 | 2 |
| *Linum usitatissimum* | *LusXB31* | Lus10009674 | 23140043 | 491 | 53142 | 7.51 | 2 |
| *Linum usitatissimum* | *LusXB32* | Lus10027441 | 23144498 | 505 | 54696 | 7.67 | 5 |
| *Linum usitatissimum* | *LusXB33* | Lus10016561 | 23144850 | 453 | 48935 | 7.14 | 6 |
| *Linum usitatissimum* | *LusXB34* | Lus10019974 | 23156115 | 514 | 55835 | 7.18 | 5 |
| *Linum usitatissimum* | *LusXB35* | Lus10040829 | 23157710 | 380 | 41006 | 8.42 | 4 |
| *Linum usitatissimum* | *LusXB36* | Lus10005754 | 23169829 | 506 | 54419 | 6.71 | 5 |
| *Linum usitatissimum* | *LusXB37* | Lus10009035 | 23173345 | 497 | 53697 | 7.52 | 2 |
| *Linum usitatissimum* | *LusXB38* | Lus10015503 | 23173604 | 587 | 64822 | 8.35 | 5 |
| *Malus domestica* | *MdoXB31* | MDP0000766001 | 22621860 | 444 | 47264 | 6.56 | 5 |
| *Malus domestica* | *MdoXB32* | MDP0000132810 | 22636222 | 613 | 66727 | 7.21 | 4 |
| *Malus domestica* | *MdoXB33* | MDP0000285560 | 22653390 | 549 | 59202 | 6.61 | 3 |
| *Malus domestica* | *MdoXB34* | MDP0000211804 | 22661488 | 504 | 55683 | 8.36 | 3 |
| *Malus domestica* | *MdoXB35* | MDP0000867915 | 22682323 | 513 | 55156 | 6.49 | 5 |
| *Mimulus guttatus* | *MguXB31* | mgv1a006609m | 17674427 | 438 | 47640 | 7.52 | 2 |
| *Mimulus guttatus* | *MguXB32* | mgv1a008790m | 17678795 | 363 | 39110.82 | 8.21 | 4 |
| *Mimulus guttatus* | *MguXB33* | mgv1a004626m | 17684097 | 518 | 55632.46 | 6.42 | 5 |
| *Mimulus guttatus* | *MguXB34* | mgv1a005221m | 17686856 | 494 | 53320.55 | 7.18 | 5 |
| *Mimulus guttatus* | *MguXB35* | mgv1a006594m | 17693158 | 438 | 47256.76 | 5.56 | 2 |
| *Mimulus guttatus* | *MguXB36* | mgv1a025493m | 17695899 | 495 | 53212.91 | 8.46 | 5 |
| *Medicago truncatula* | *MtrXB31* | AC146807_5.1 | 17431890 | 514 | 55691.57 | 6.41 | 5 |
| *Medicago truncatula* | *MtrXB32* | Medtr8g127710.1 | 17483567 | 510 | 55135.76 | 7.72 | 5 |
| *Oryza sative* | *XBOS33* | LOC_Os10g37730.1 | 21885116 | 521 | 55562.22 | 5.88 | 5 |
| *Oryza sative* | *XBOS35* | LOC_Os08g15840.1 | 21887507 | 496 | 53082.14 | 8.86 | 6 |
| *Oryza sative* | *XBOS34* | LOC_Os07g26490.1 | 21899951 | 514 | 54729.1 | 5.96 | 3 |
| *Oryza sative* | *XBOS31* | LOC_Os01g74320.1 | 21907276 | 447 | 47701.42 | 8.26 | 5 |
| *Oryza sative* | *XBOS37* | LOC_Os03g16780.1 | 21913163 | 466 | 49164.71 | 7.86 | 6 |
| *Oryza sative* | *XBOS32* | LOC_Os02g54860.1 | 21922926 | 497 | 53617.3 | 6.72 | 6 |
| *Oryza sative* | *XBOS36* | LOC_Os06g03800.1 | 21931521 | 421 | 43456.5 | 6.30 | 5 |
| *Oryza sative* | *XB3* | LOC_Os05g02130.1 | 21943354 | 451 | 48202.84 | 6.77 | 6 |
| *Physcomitrella patens* | *PpaXB31* | Pp1s60_316V6.1 | 18049062 | 477 | 50965.04 | 5.31 | 4 |
| *Physcomitrella patens* | *PpaXB32* | Pp1s256_42V6.1 | 18051072 | 462 | 49893.25 | 6.26 | 5 |
| *Physcomitrella patens* | *PpaXB33* | Pp1s133_21V6.1 | 18061636 | 497 | 53317.41 | 5.33 | 4 |
| *Physcomitrella patens* | *PpaXB34* | Pp1s196_117V6.1 | 18062455 | 515 | 56051.07 | 8.9 | 5 |
| *Physcomitrella patens* | *PpaXB35* | Pp1s55_144V6.1 | 18063155 | 563 | 60754.23 | 8.55 | 5 |
| *Physcomitrella patens* | *PpaXB36* | Pp1s13_27V6.1 | 18064758 | 560 | 60251.06 | 8.51 | 5 |
| *Physcomitrella patens* | *PpaXB37* | Pp1s137_113V6.1 | 18072751 | 482 | 51585.63 | 5.77 | 4 |
| *Prunus persica* | *PpeXB31* | ppa005750m | 17643116 | 446 | 48322.26 | 7.25 | 5 |
| *Prunus persica* | *PpeXB32* | ppa004764m | 17649648 | 493 | 53422.93 | 7.84 | 3 |
| *Prunus persica* | *PpeXB33* | ppa005321m | 17649649 | 468 | 50262.26 | 5.97 | 3 |
| *Prunus persica* | *PpeXB34* | ppa005792m | 17662929 | 444 | 47487.36 | 6.53 | 4 |
| *Prunus persica* | *PpeXB35* | ppa004397m | 17652671 | 513 | 54994.92 | 6.39 | 5 |
| *Prunus persica* | *PpeXB36* | ppa004480m | 17654011 | 509 | 55041.67 | 8.20 | 5 |
| *Populus trichocarpa* | *PtrXB31* | POPTR_0006s19070.1 | 18211693 | 508 | 54949.79 | 7.92 | 5 |
| *Populus trichocarpa* | *PtrXB32* | POPTR_0018s10750.1 | 18215535 | 510 | 55293.17 | 8.02 | 5 |
| *Populus trichocarpa* | *PtrXB33* | POPTR_0009s03530.1 | 18227180 | 448 | 48339.22 | 8.39 | 5 |
| *Populus trichocarpa* | *PtrXB34* | POPTR_0012s13410.1 | 18229596 | 512 | 55237.96 | 6.19 | 5 |
| *Populus trichocarpa* | *PtrXB35* | POPTR_0015s13360.1 | 18233168 | 512 | 55138.01 | 6.16 | 6 |
| *Populus trichocarpa* | *PtrXB36* | POPTR_0001s24570.1 | 18235718 | 443 | 47953.67 | 6.63 | 6 |
| *Populus trichocarpa* | *PtrXB37* | POPTR_0010s08150.1 | 18240068 | 445 | 48182.06 | 5.91 | 2 |
| *Populus trichocarpa* | *PtrXB38* | POPTR_0010s08160.1 | 18241678 | 456 | 49500.77 | 5.62 | 3 |
| *Populus trichocarpa* | *PtrXB39* | POPTR_0008s16740.1 | 18248213 | 476 | 51264.24 | 5.67 | 2 |
| *Panicum virgatum* | *PviXB31* | Pavirv00002765m | 23768249 | 518 | 55418.04 | 5.97 | 5 |
| *Panicum virgatum* | *PviXB32* | Pavirv00025380m | 23780210 | 513 | 54648.27 | 6.67 | 3 |
| *Panicum virgatum* | *PviXB33* | Pavirv00044808m | 23780540 | 448 | 47776.27 | 8.42 | 6 |
| *Panicum virgatum* | *PviXB34* | Pavirv00066820m | 23780887 | 449 | 47901.25 | 8.10 | 6 |
| *Panicum virgatum* | *PviXB35* | Pavirv00003155m | 23782333 | 497 | 53666.21 | 6.64 | 5 |
| *Panicum virgatum* | *PviXB36* | Pavirv00019355m | 23793454 | 484 | 51807.45 | 8.82 | 5 |
| *Panicum virgatum* | *PviXB37* | Pavirv00039060m | 23796042 | 453 | 48410.16 | 7.23 | 5 |
| *Panicum virgatum* | *PviXB38* | Pavirv00008792m | 23798180 | 453 | 48421.16 | 6.99 | 5 |
| *Panicum virgatum* | *PviXB39* | Pavirv00049144m | 23804478 | 484 | 51817.56 | 8.85 | 5 |
| *Panicum virgatum* | *PviXB310* | Pavirv00068522m | 23809415 | 420 | 42996.92 | 6.30 | 5 |
| *Panicum virgatum* | *PviXB311* | Pavirv00028028m | 23810730 | 513 | 54633.01 | 6.06 | 5 |
| *Panicum virgatum* | *PviXB312* | Pavirv00061839m | 23817852 | 403 | 43793.89 | 6.11 | 5 |
| *Panicum virgatum* | *PviXB313* | Pavirv00008053m | 23823939 | 511 | 54479.07 | 6.52 | 3 |
| *Panicum virgatum* | *PviXB314* | Pavirv00000305m | 23825051 | 420 | 43216.29 | 6.40 | 5 |
| *Ricinus communis* | *RcoXB31* | 28597.m000123 | 16801194 | 511 | 55531.5 | 7.46 | 5 |
| *Ricinus communis* | *RcoXB32* | 30147.m014285 | 16820296 | 513 | 55315.32 | 6.27 | 5 |
| *Sorghum bicolor* | *SbiXB31* | Sb01g031230.1 | 1952852 | 521 | 55549.13 | 6.02 | 5 |
| *Sorghum bicolor* | *SbiXB32* | Sb01g039610.1 | 1953900 | 449 | 48063.5 | 8.42 | 6 |
| *Sorghum bicolor* | *SbiXB33* | Sb02g010610.1 | 1956604 | 517 | 55380.95 | 6.23 | 3 |
| *Sorghum bicolor* | *SbiXB34* | Sb03g047430.1 | 1964819 | 485 | 51264.12 | 6.77 | 5 |
| *Sorghum bicolor* | *SbiXB35* | Sb04g035590.1 | 1968427 | 498 | 53846.74 | 6.93 | 5 |
| *Sorghum bicolor* | *SbiXB36* | Sb07g008820.1 | 1975551 | 484 | 51907.96 | 8.95 | 5 |
| *Sorghum bicolor* | *SbiXB37* | Sb09g001370.1 | 1979466 | 466 | 49424.12 | 6.64 | 6 |
| *Sorghum bicolor* | *SbiXB38* | Sb10g001750.1 | 1982347 | 407 | 42103.15 | 6.59 | 5 |
| *Setaria italica* | *SitXB31* | Si035733m | 19680827 | 443 | 47115.58 | 8.41 | 6 |
| *Setaria italica* | *SitXB32* | Si034630m | 19684470 | 520 | 55465.08 | 6.21 | 5 |
| *Setaria italica* | *SitXB33* | Si034656m | 19684471 | 512 | 54513.95 | 6.20 | 5 |
| *Setaria italica* | *SitXB34* | Si017011m | 19686900 | 497 | 53732.52 | 7.17 | 6 |
| *Setaria italica* | *SitXB35* | Si013655m | 19692078 | 484 | 51920.65 | 8.90 | 6 |
| *Setaria italica* | *SitXB36* | Si021653m | 19702003 | 456 | 48625.49 | 6.98 | 6 |
| *Setaria italica* | *SitXB37* | Si006533m | 19704450 | 418 | 43023.96 | 6.21 | 5 |
| *Setaria italica* | *SitXB38* | Si006797m | 19704452 | 348 | 36070.08 | 6.19 | 4 |
| *Setaria italica* | *SitXB39* | Si029529m | 19712709 | 514 | 54462 | 6.52 | 3 |
| *Selaginella moellendorffii* | *SmoXB31* | 441660 | 15409448 | 591 | 63507.39 | 5.97 | 6 |
| *Selaginella moellendorffii* | *SmoXB32* | 141633 | 15411241 | 448 | 48806.27 | 9.5 | 4 |
| *Selaginella moellendorffii* | *SmoXB33* | 81884 | 15411483 | 476 | 51161.67 | 7.13 | 4 |
| *Selaginella moellendorffii* | *SmoXB34* | 175493 | 15420739 | 532 | 56824.7 | 6.56 | 5 |
| *Selaginella moellendorffii* | *SmoXB35* | 86281 | 15421353 | 401 | 43059.34 | 8.28 | 5 |
| *Thellungiella halophila* | *ThaXB31* | Thhalv10016642m | 20179909 | 456 | 49194.44 | 8.33 | 5 |
| *Thellungiella halophila* | *ThaXB32* | Thhalv10016854m | 20179910 | 360 | 38810.41 | 8.09 | 3 |
| *Thellungiella halophila* | *ThaXB33* | Thhalv10020731m | 20183649 | 446 | 48321.23 | 8.48 | 2 |
| *Thellungiella halophila* | *ThaXB34* | Thhalv10025615m | 20193825 | 348 | 38593.26 | 6.46 | 2 |
| *Thellungiella halophila* | *ThaXB35* | Thhalv10013287m | 20203984 | 512 | 55209.97 | 6.31 | 5 |
| *Thellungiella halophila* | *ThaXB36* | Thhalv10013312m | 20204616 | 505 | 54205.64 | 8.51 | 5 |
| *Vitis vinifera* | *VviXB31* | GSVIVT01010599001 | 17822713 | 513 | 55095.96 | 6.34 | 5 |
| *Vitis vinifera* | *VviXB32* | GSVIVT01020545001 | 17829875 | 498 | 53174.48 | 6.56 | 4 |
| *Vitis vinifera* | *VviXB33* | GSVIVT01023777001 | 17832102 | 510 | 55213.04 | 8.31 | 5 |
| *Vitis vinifera* | *VviXB34* | GSVIVT01024983001 | 17833067 | 396 | 42537.62 | 6.30 | 6 |
| *Vitis vinifera* | *VviXB35* | GSVIVT01034187001 | 17839751 | 445 | 47836.82 | 7.21 | 6 |
| *Zea mays* | *ZmaXB31* | GRMZM2G142345_T01 | 20823012 | 409 | 41889.82 | 6.27 | 5 |
| *Zea mays* | *ZmaXB32* | GRMZM2G069162_T04 | 20824833 | 495 | 52451.34 | 6.86 | 6 |
| *Zea mays* | *ZmaXB33* | GRMZM2G016668_T01 | 20826734 | 420 | 44967.36 | 7.26 | 5 |
| *Zea mays* | *ZmaXB34* | GRMZM2G133396_T01 | 20835123 | 518 | 55399.07 | 6.23 | 3 |
| *Zea mays* | *ZmaXB35* | GRMZM2G006973_T01 | 20838526 | 437 | 44856.06 | 8.20 | 5 |
| *Zea mays* | *ZmaXB36* | GRMZM2G103270_T01 | 20838684 | 447 | 48026.59 | 8.60 | 6 |
