# Supplementary material for: Global Analysis of Ankyrin Repeat Domain C3HC4-Type RING Finger Gene Family in Plants
Source: PLoS One. 2013 Mar 13;8(3):e58003. doi: 10.1371/journal.pone.0058003 (PMC3596331; doi:10.1371/journal.pone.0058003)
Supplement: Table S2 — Number and consensus of each group type C3HC4-RING domain identified in plants. (DOC) [file pone.0058003.s006.doc]

Table S2 Number and consensus of each group type C3HC4-RING domain identified in plants.

| Types | Numbers | Consensus |
| --- | --- | --- |
| Group Ⅰ | 81 | C-X2-C-X11-C-X1-H-X2-C-X2-C-X19~23-C-X2-C |
| Group Ⅱ | 54 | C-X2-C-X11-C-X1-H-X2-C-X2-C-X21~24-C-X2-C |
| Group Ⅲ | 52 | C-X2-C-X11-C-X1-H-X2-C-X2-C-X10-C-X2-C |
